# Anastomotic Leak and Perioperative Outcomes of Esophagectomy for Esophageal Cancer during the COVID-19 Pandemic: A Systematic Review and Meta-Analysis

**DOI:** 10.3390/medicina60010031

**Published:** 2023-12-24

**Authors:** Georgios Geropoulos, Stavros Moschonas, Georgios Fanariotis, Aggeliki Koltsida, Nikolaos Madouros, Evgenia Koumadoraki, Kontantinos Katsikas Triantafyllidis, Konstantinos S. Kechagias, Georgios Koimtzis, Dimitrios Giannis, Athanasios Notopoulos, Efstathios T. Pavlidis, Kyriakos Psarras

**Affiliations:** 1 Second Surgical Propedeutic Department, Hippocration Hospital, Aristotle University of Thessaloniki, 54124 Thessaloniki, Greece; pavlidis.md@gmail.com; 2 Surgery Working Group, Society of Junior Doctors, 15123 Athens, Greece; mschstavros@gmail.com (S.M.); aggeliki.koltsida@gmail.com (A.K.); evgenia.koumadoraki@nhs.net (E.K.); 3 Department of Nutrition and Dietetics, Royal Marsden Hospital, London SW3 6JJ, UK; k.katsikas-triantafyllidis18@alumni.imperial.ac.uk; 4 Department of Obstetrics and Gynaecology, The Hillingdon Hospitals NHS Foundation Trust, Uxbridge UB8 3NN, UK; 5 Department of General Surgery, University Hospital of Wales, Cardiff and Vale University Health Board, Cardiff CF14 4XW, UK; georgios.koimtzis@wales.nhs.uk; 6 Department of Surgery, Flushing Hospital Medical Center, Flushing, NY 11355, USA; dimitrisgiannhs@gmail.com; 7 Department of Nuclear Medicine, Hippocration Hospital, Aristotle University of Thessaloniki, 54124 Thessaloniki, Greece; nuclearmed@ippokratio.gr

**Keywords:** esophageal cancer, COVID-19, esophagectomy, perioperative outcomes, post-operative outcomes, systematic review, meta-analysis

## Abstract

*Background and Objectives*: The coronavirus disease-2019 (COVID-19) pandemic influenced the healthcare system tremendously, as well as the number of elective surgical procedures worldwide. The aim of this study is to investigate the COVID-19 pandemic’s impact on esophagectomies. *Materials and Methods*: The MEDLINE (via PubMed), Cochrane Library, and Google Scholar bibliographical databases were systematically searched. Original clinical studies investigating the outcomes of esophageal cancer surgery during the COVID-19 pandemic were deemed eligible. After exclusion criteria were applied, eight studies were considered eligible for inclusion. *Results*: Eight studies with non-overlapping populations, reporting on patients undergoing esophagectomy for resectable esophageal cancer during the COVID-19 pandemic, were included in our analysis, with a total of 18548 patients. Background characteristics for age, lung disease, smoking history as well as Body Mass Index and age were equal among the groups. The background of diabetes presented a statistically significant difference among the groups. Perioperative outcomes like reoperation rates, the length of intensive care unit stay, or readmission rates were not significantly increased during the pandemic. The 30-day readmission, and 30- and 90-day mortality were not affected either. The length of hospital stay was significantly lower in the non-pandemic period. *Conclusions*: The results of our study support the evidence that in the context of the COVID-19 pandemic, esophageal cancer operations took place safely and effectively, similarly to the standards of the non-COVID-19 era.

## 1. Introduction

The coronavirus disease-2019 (COVID-19) pandemic has significantly affected all aspects of the global healthcare system [1]. The year 2022 was marked by the emergence of the Omicron variant as it spread and became the dominant variant worldwide. Approximately 360 million cases were reported to WHO in 2022, representing more than half of COVID-19 cases reported since the start of the pandemic, and more than during the previous two years combined [2]. The number of positive tests almost tripled in 2022, as it rose to 20% from 7% during the first two years of the pandemic. In the same year, approximately 1.2 million people are estimated to have died from COVID-19, representing one in five deaths in total. Hopefully, as WHO reported in January 2023, the number of deaths is declining overall, with between 8000 and 10,000 deaths being reported per week.

In this setting, during the first wave of the pandemic, millions of elective surgical procedures were canceled worldwide, in order to provide better care to COVID-19 patients and generally support the wider response [3]. In fact, it is estimated that, during the peak 12 weeks of disruption due to the coronavirus pandemic, the cancellation rate would be 72.3 per cent [4]. Profoundly, canceling or postponing elective surgery on this scale could potentially have an important impact on surgical patients and, consequently, catastrophic results for health systems worldwide [5]. Patients with esophageal malignancies develop multiple cancer-related complications, like pain, malnutrition, and poor performance status, with dysphagia caused by local tumor progression being one of the most frequent symptoms in patients with advanced disease. Clinicians should be alerted about dysphagia, being aware about other causes like stroke or foreign bodies, and refer to cancer centers early if other red-flag symptoms exist (weight loss, progressive dysphagia, or evidence of metastatic disease). 

Furthermore, some elective surgical operations for time-sensitive cases continued, with a prioritization of patients whose underlying cancer was deemed resectable but would be at risk for progression, and patients with no other alternative effective treatment modalities. Indeed, COVID-19-free surgical pathways in both elective and major acute hospitals were established, in which elective operating rooms, critical care, and inpatient ward areas were not shared with COVID-19 patients [6]. The purpose of this study is to systematically retrospectively analyze the impact of the coronavirus pandemic on upper GI surgeries and especially on esophagectomies for esophageal cancer to assess any differences in perioperative and post-operative outcomes and delays. 

## 2. Materials and Methods

### 2.1. Study Design and Inclusion/Exclusion Criteria

This systematic review and meta-analysis project was conducted according to the PRISMA (Preferred Reporting Items for Systematic Reviews and Meta-analyses) guidelines and in line with the protocol developed and agreed a priori by all authors [7]. Studies investigating esophagectomy outcomes during the COVID-19 pandemic and non-pandemic time periods were deemed eligible for analysis. As a result, two groups of patients were involved in this study: one group of patients that had an esophagectomy during the COVID-19 pandemic and one group that had an esophagectomy during the non-pandemic era. Exclusion criteria for the present systematic review were the following: (i) articles published in languages other than English; (ii) narrative, systematic, and meta-analysis reviews; (iii) case reports, errata, comments, perspectives, letters to the editor, and editorials that did not provide any primary patient data for the COVID-19 and non-COVID-19 esophagectomy period; (iv) published abstracts with no available full text; and (v) non-comparative studies (fewer than two study arms). No publication date, sample size restrictions, or any other search filters were applied.

### 2.2. Search Strategy

Eligible studies were identified via a search through the MEDLINE (via PubMed), Cochrane Library, and Scopus databases (end-of-search date: 1 August 2023) carried out by two independent researchers (GF and SM). The algorithm used for every search was the combination of the following keywords with all available synonyms: (esophagectomy) (esophageal cancer) AND (COVID-19). Any disagreements during the screening process were resolved by a third reviewer (GG). The reference lists and all previously published systematic reviews were thoroughly searched for missed studies eligible for inclusion based on the “snowball” methodology [8].

### 2.3. Data Extraction

A standardized, pre-piloted form was used for data tabulation and extraction. Two reviewers (MF and PG) extracted the data independently, and any disagreements were identified and resolved by a third reviewer (GG). We extracted the following data from the included studies: (i) study characteristics (first author, year of publication, study design, study center, country, study period, number of patients), (ii) patient characteristics (BMI, smoking, age, American Society of Anesthesiology (ASA) score of III), (iii) mortality outcomes, and iv) long-term post-operative outcomes.

### 2.4. Risk of Bias Assessment

The Methodological Index for Non-Randomized Studies (MINORS) was used for the risk of bias assessment of observational studies [9]. MINORS is a valid instrument designed to assess the methodological quality of non-randomized studies, whether comparative or non-comparative. MINORS’ domains are scored as 0 if they are not reported, 1 when they have been reported but with inadequate details, and 2 when they have been reported while providing adequate information. The global ideal score is 16 for non-comparative studies; MINORS < 6 is considered as high risk of bias, while MINORS between 6 and 9 is considered as moderate risk of bias [9].

### 2.5. Statistical Analysis

The available data were handled according to the principles stated in the Cochrane Handbook [7]. Data on outcomes of interest were summarized and analyzed cumulatively. Categorical variables were reported as number of events among the total cases. Based on the extracted data, odds ratio (OR) and 95% confidence interval (CI) were calculated by means of 2 × 2 tables for each categorical event; OR > 1 indicated that the trait was more frequently present in the COVID-19 pandemic group. This is depicted in the forest plot for each separate variable. Between-study heterogeneity was assessed by estimating the I2 statistic. High heterogeneity was confirmed with a significance level of *p* < 0.05 and I2 ≥ 50%. The random-effects model was used to calculate the pooled effect when heterogeneity was high, while the fixed-effects model was used when low heterogeneity was encountered. All statistical analyses and forest plot generation were performed with the use of Reviewer Manager 5.4.1 software (Review Manager (RevMan) [Computer Program]. Version 5.4.1, Copenhagen: The Nordic Cochrane Centre, Denmark, The Cochrane Collaboration, 2020).

## 3. Results

**Figure 1:**
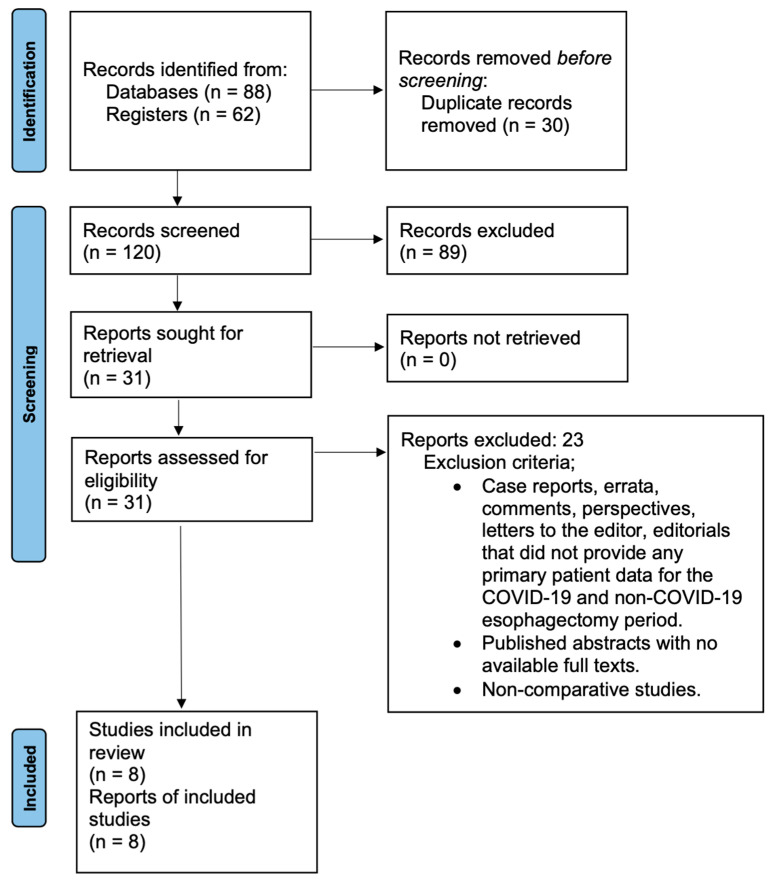
PRISMA 2020 flow diagram.

A total of 150 studies were initially identified through our systematic literature search. After adjusting for duplicates, 30 of them were removed and the records of the remaining 120 underwent the screening process. As a result, 89 of them were discarded as they proved to be irrelevant, and the full text of the remaining 31 was retrieved. All these data are available in the PRISMA flow diagram (Figure 1).

Table 1 summarizes the key characteristics of the included studies. The included studies’ participants ranged from 70 to 17,351, with a total of 14,301 subjects from the control group and 4247 subjects treated during the COVID-19 pandemic. The studies were conducted in various geographic regions, including Europe, the United States of America, and Japan. All studies used a case–control study design [1,10,11,12,13,14,15,16].

The overall risk of bias was low, apart from one study which was determined to have moderate risk (Table 2). The most measured outcomes were 30-day mortality, the risk of grade IV Clavien–Dindo complications, and the total length of hospital stay. However, not all studies measured the same outcomes.

### 3.1. Baseline Patient Characteristics

**Figure 2:**
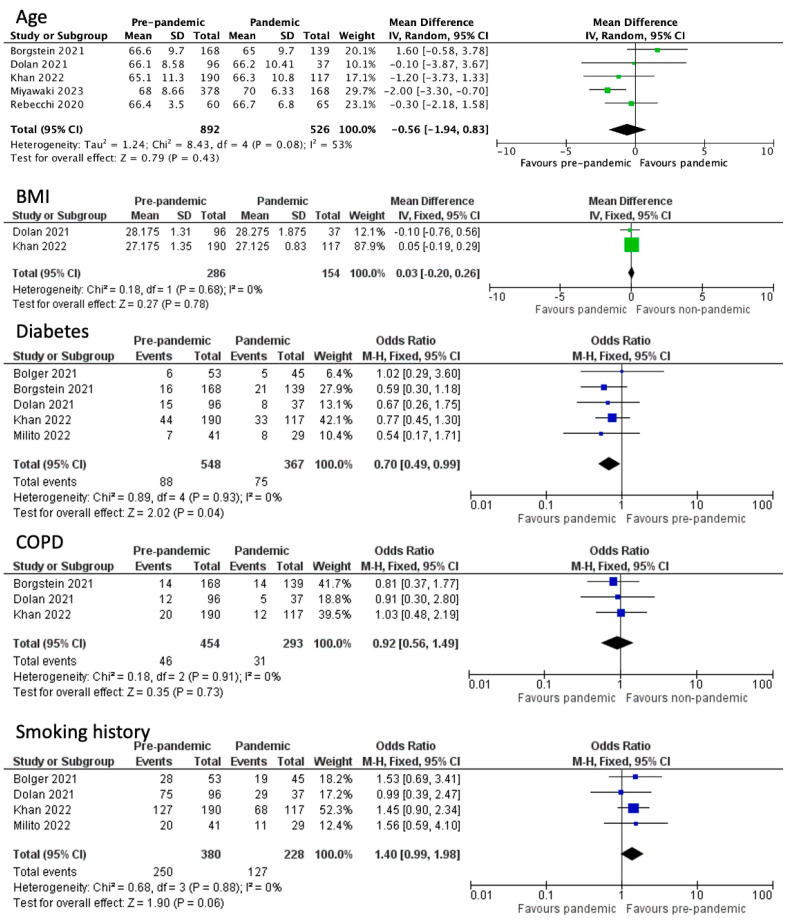
Patient basic characteristics [1,10,11,12,13,14,16].

Patient characteristics did not seem to have a statistically significant difference among the two groups. Patients with COPD (OR 0.92, CI 95% 0.92 to 1.49, *p* = 0.73, I^2^ = 0%), smoking history (OR 1.40, CI 95% 0.99 to 1.98, *p* = 0.06, I^2^ = 0%), and ASA score of III (OR 0.82, CI 95% 0.53 to 1.27, *p* = 0.37, I^2^ = 67%) did not differ in a statistically significant manner between the groups. The patients’ BMI did not fluctuate significantly (WMD 0.03, CI 95% −0.20 to 0.26, *p* = 0.78 I^2^ = 0%); however, as was the case for the ASA score, it was only measured in two studies [1,10,11,12]. Age did not present a significant difference either (WMD-0.56, CI 95% −1.94 to 0.83, *p* = 0.08, I^2^ = 53%). The only characteristic that exhibited a statistically significant difference was the incidence of diabetes (OR 0.70, CI 95% 0.49 to 0.99, *p* = 0.04, I^2^ = 0%), as proportionally more patients with diabetes received treatment during the pandemic [1,10,11,12,13,14,16] (Figure 2).

**Figure 3:**
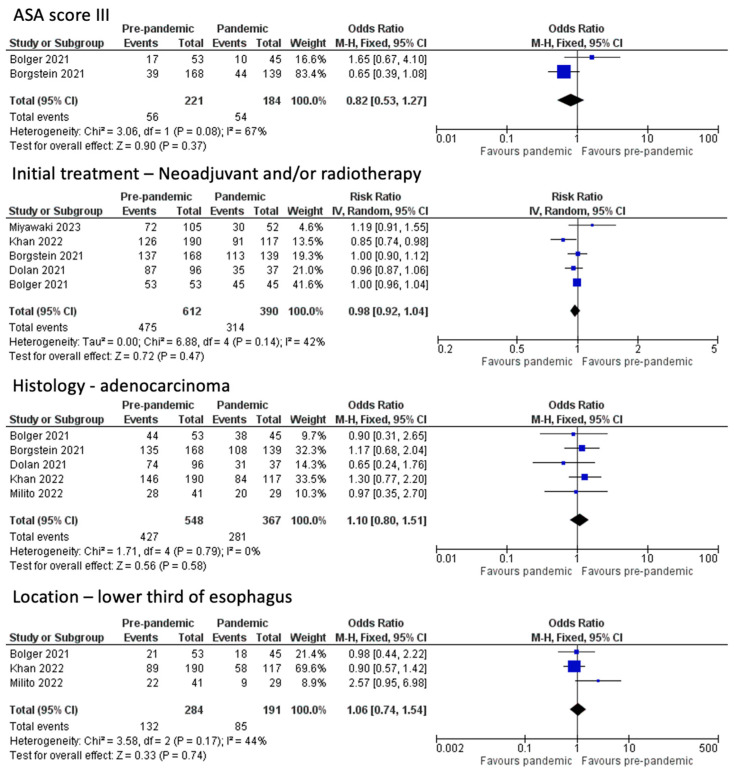
ASA score, treatment options, and tumor characteristics [1,10,11,12,14,16].

Before moving to the post-operative outcomes, we chose to measure the proportion of patients receiving initial treatment (neoadjuvant and/or radiotherapy) in the two groups, which did not show a statistically significant difference (OR 0.98, CI 95% 0.92 to 1.04, *p* = 0.47, I^2^ = 42%) [1,10,11,12,16]. In terms of histological diagnosis, the characteristics of the tumor with regard to the prevalence of adenocarcinoma (OR 1.10, CI 95% 0.80 to 1.51, *p* = 0.58, I^2^ = 0%) and its location (lower third: OR 1.06, CI 95% 0.74 to 1.54, *p* = 0.74, I^2^ = 44%; gastroesophageal junction: OR 0.87, CI 95% 0.62 to 1.22, *p* = 0.41, I^2^ = 57%) did not exhibit a statistically significant difference either [1,10,11,12,14] (Figure 3).

### 3.2. Post-Operative Outcomes

**Figure 4:**
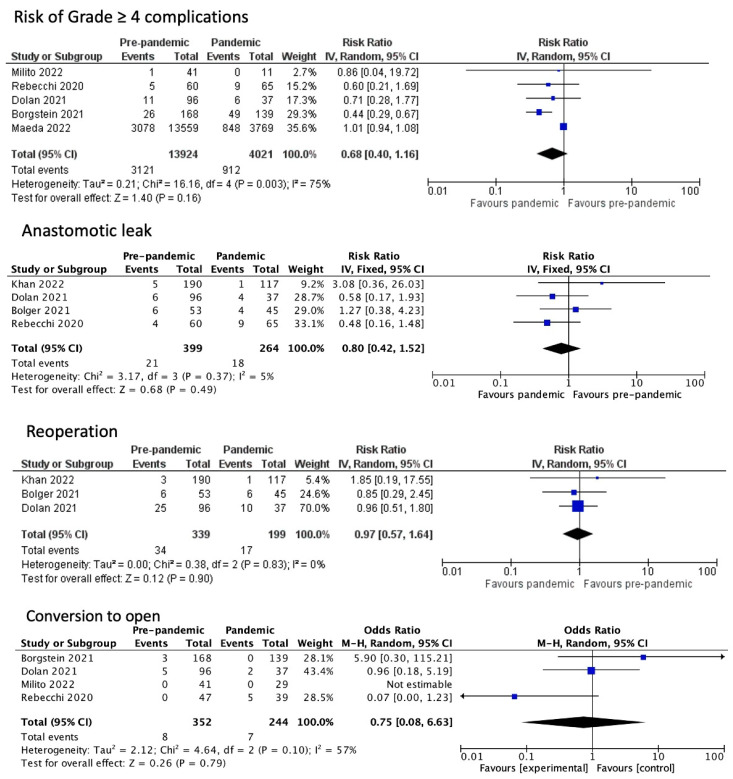
Immediate post-operative outcomes [1,10,11,12,13,14,15].

In regard to the immediate post-operative outcomes, the proportion of patients who required re-intubation (OR 0.76, CI 0.36 to 1.59, *p* = 0.76, I2 = 22%) or reoperation (OR 0.97, CI 95% 0.57 to 1.64, *p* = 0.90, I^2^ = 0%) showed no statistically significant difference. Similarly, the percentage of grade IV Clavien–Dindo post-operative complications (OR 0.68, CI 0.40 to 1.16, *p* = 0.16, I^2^ = 75%) as well as the rates of anastomotic leak (OR 0.8, CI 95% 0.41 to 1.57, *p* = 0.52 I2 = 5%) did not differ. These data are also coherent with the rates of conversion to an open surgical procedure (OR 0.67, CI 0.2 to 1.82, *p* = 0.43, I^2^ = 57%), which also showed no statistically significant difference (Figure 4).

**Figure 5:**
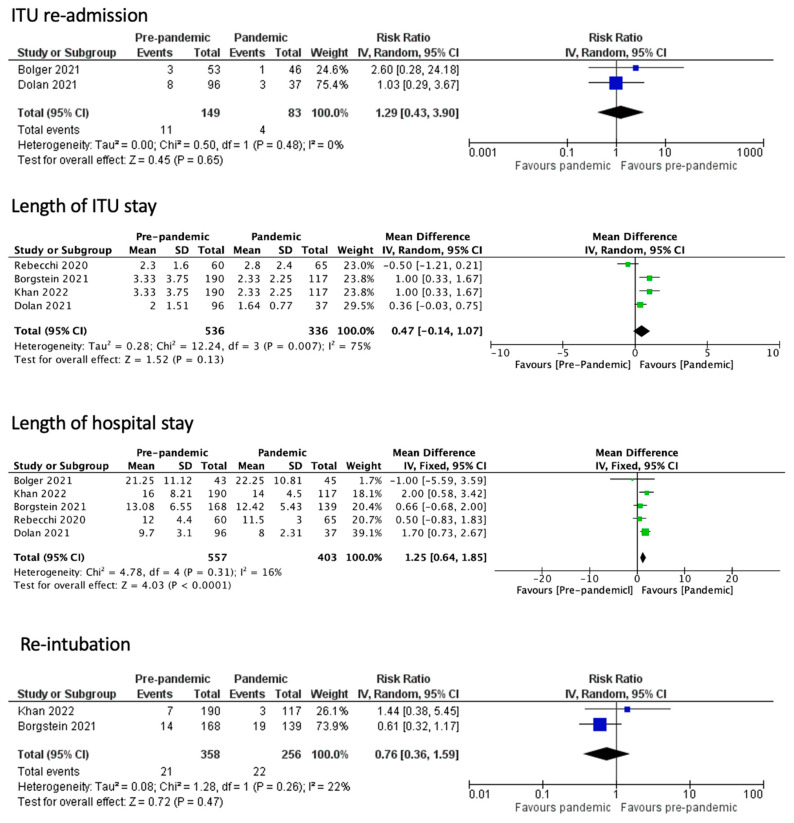
Perioperative outcomes [1,10,11,12,13].

**Figure 6:**
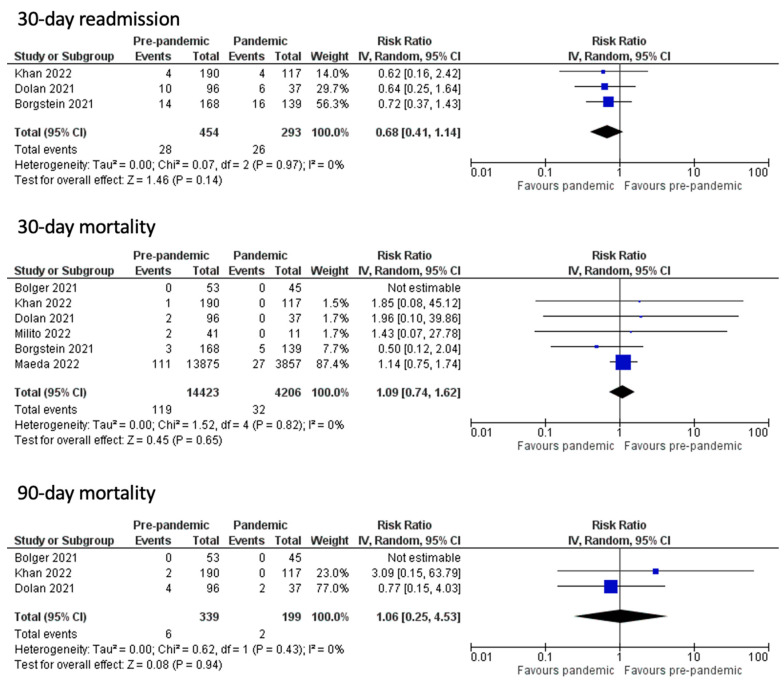
Readmission and mortality outcomes [1,10,11,12,14,15].

**Figure 7:**
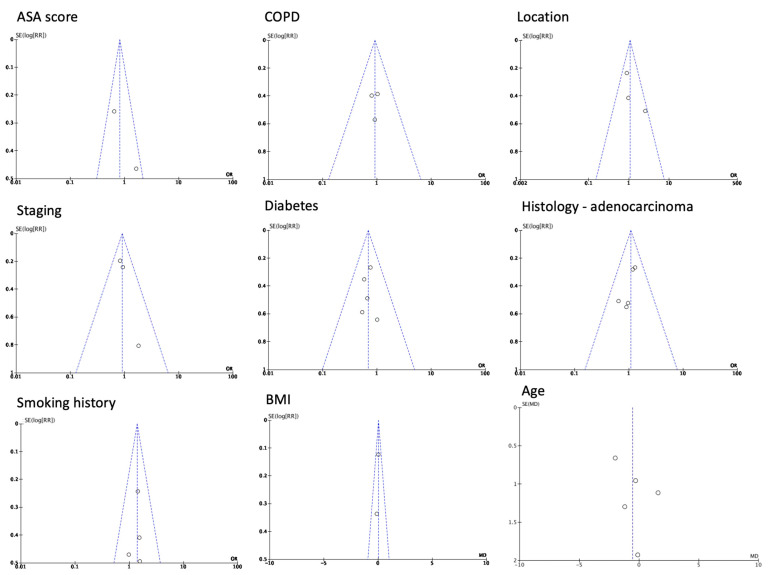
Funnel plots of basic patient characteristic outcomes.

**Figure 8:**
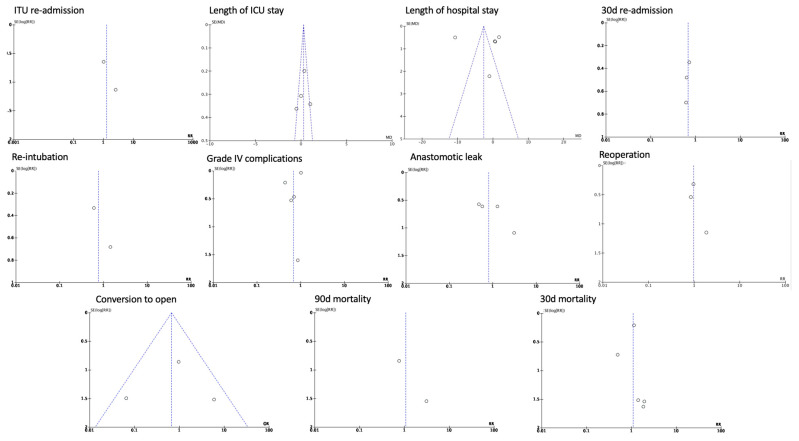
Funnel plots of immediate and long-term outcomes.

The effects of the immediate post-operative period carry on to the rest of the hospital stay of the patients, as the rates for ITU readmission (RR 1.29, 95% CI 0.43 to 3.90, *p* = 0.65 I^2^ = 0%) and the overall length of the ITU hospitalization (WMD 0.47, CI 95% − 0.14 to 1.07, *p* = 0.13, I^2^ =75%) had no statistically significant differentiation between the groups. However, the only measured outcome that showed statistically significant difference was the overall length of hospitalization (WMD 1.25, CI 95% 0.64 to 1.85, *p* < 0.001, I^2^ = 16%), where the non-pandemic group had a significantly lower overall length of hospitalization. Long-term post-operative outcomes, including the 30-day readmission (OR 0.68, CI 95% 0.41 to 1.14, *p* = 0.14, I^2^ = 0%), the 30-day mortality (OR 1.09, CI 95% 0.74 to 1.62, *p* = 0.65, I^2^ = 0%), and the 90-day mortality rates (OR 1.06, CI 95% 0.25 to 4.53, *p* = 0.94, I^2^ = 0%) showed no statistically significant difference (Figure 5 and Figure 6). The funnel plot analysis of the aforementioned variables is depicted in Figure 7 and Figure 8.

## 4. Discussion

This systematic review and meta-analysis project attempted to study the effects of the COVID-19 pandemic on patients undergoing esophagectomy. The aim of this study is to compare patient outcomes undergoing a surgical treatment of esophageal cancer during the COVID-19 pandemic versus the non-COVID-19 pandemic era. More specifically, we found that most outcomes (30-day mortality, 30-day readmission rate, 90-day mortality, the initial type of treatment offered, re-intubation, conversion to open surgical procedure, reoperation, anastomotic leak, ITU readmission, graded IV Clavien–Dindo post-operative complications, and the length of ITU hospitalization) did not differ significantly between the two groups. The length of total hospitalization as well as the diabetes were the only patient characteristics that had a statistically significant differentiation among the ones that were included in this study. These results support the fact that during the pandemic, safe esophagectomy practice could exist. It seems that policies taken by different hospitals around the world led to the safe surgical management of esophageal malignancies. 

The management of esophageal cancer includes surgery, radiation therapy, chemotherapy, immunotherapy, or a combination of these treatment strategies [17]. The absence of a statistically significant difference in the type of initial therapy modalities offered for esophageal cancer patients during the COVID-19 pandemic, compared to the treatment modalities administered to another group of patients a year before the pandemic, is a strong indicator of a high-quality organizational effort and the persistence of healthcare staff to continue to provide a quality of service as close to the established standard of care as possible. While the pandemic did have a dampening effect on the activities of many surgical institutes and intensive care units, some centers for esophageal cancer modified their protocols to be able to run as a green hub [10,18]. As a result, their personnel were able to act as close as possible to the pre-pandemic standard of care. Furthermore, the similarity in esophageal cancer treatment rates before and during the COVID-19 pandemic sheds light on the prioritization that was ensured for this group of high-risk patients. All the aforementioned factors may have contributed to the fact that esophagectomy did not meet the fate of other elective procedures during the COVID-19 pandemic [19]. 

Basic patient characteristics (BMI, COPD, smoking history, and ASA score of III), as well as histological features (adenocarcinoma, and location at the lower third of the esophagus), did not exhibit a statistically significant difference apart from age and diabetes. In the case of chronological age, it was more common for the patients receiving surgical treatment before the pandemic to be younger. Although no clear explanation exists, a potential reason for having younger patients in the non-pandemic era may be attributed to lockdown policies where young people seek solely emergency care rather than preventive medicine. On the other hand, old people tend to have a more extensive disease at the initial diagnosis and become significantly symptomatic, so they may seek emergency care more frequently [20]. Similarly, patients with esophageal cancer and diabetes constitute a group whose physiology and characteristics play a significant part in the decision-making process for a surgical oncology operation. Combining this with the fact that the presence of diabetes acts as an independent factor for decreased pathologic complete response, as Alvarado et al. showed in a recent study, these factors could justify the prioritization of diabetic patients to receive surgical treatment for esophageal cancer [21]. However, further research is deemed necessary to clarify the age and diabetes differences presented in this study. 

Adding to that, the statistical data of post-operative mortality at 30 and 90 days did not display any remarkable difference between the pre-pandemic and pandemic settings. Moreover, it is likely that the SARS-CoV-2 infection itself did not affect the early post-operative outcomes of the esophageal cancer patients undergoing esophagectomy. However, admittedly, many institutions had to implement different strategies regarding patient selection. One could raise an argument about the unknown number of patients with undiagnosed esophageal cancer who did not seek medical advice in a timely manner due to hesitancy in accessing healthcare services because of the fear of COVID-19 transmission. Thus, a group of this feeble high-risk population, perhaps, did not undergo esophageal resection even though such a population would be eligible for esophagectomy. It is possible that this phenomenon could obscure the true effect of SARS-CoV-2 infection on the post-operational prognosis of esophageal cancer patients.

While most of the outcomes that were included in this meta-analysis had no statistical significance, only the total length of hospitalization was favored during the time of the pandemic. All other metrics associated with post-operative complications (30-day mortality and readmission, 90-day mortality, the initial type of treatment offered, re-intubation, conversion to open surgical procedure, reoperation, anastomotic leak and ITU readmission, graded IV Clavien–Dindo post-operative complications, and the length of ITU hospitalization) were found to have no statistically significant difference between the groups. Our study concludes that the total length of hospital stay during the COVID-19 pandemic is significantly increased. However, other case series report a significant decrease in the length of hospital stay during the COVID-19 pandemic [22,23,24]. One important component of the decreased hospital stay presented by these studies could be assigned to improved infection control measures during the pandemic and the need for early discharge [25,26]. A reduced patient volume during the pandemic could have provided the chance for individualized care to patients, potentially leading to a different recovery pathway and better outcomes for the patients [1,10,27]. 

Nevertheless, the challenging question of whether the SARC-CoV-2 infection itself presents perioperative risks for patients undergoing esophagectomy, as no patient in the included studies contracted COVID-19 during the post-operative period, is yet to be determined. However, in the included studies, there is also no evidence that it can influence them negatively, for example via anastomotic leakage [12]. With regard to more severe complications requiring ITU readmission, re-intubation, or reoperation, they also were not noted to differentiate in a statistically significant manner between the groups. As far as re-intubation is concerned, it could be attributed to complications such as pneumonia [5], respiratory failure, or anastomotic leak, which also did not differ between the two groups, or to pre-existing factors such as respiratory disease and prolonged surgery duration. The fact that severe complications were avoided, to the point that more intensive methods of care were not administered further, proves both the non-significant changes between the populations studied and the quality of care provided. However, these results should be interpreted with caution as no patients actually had a COVID-19 infection. 

It must be mentioned that our meta-analysis does have some limitations. As more and more studies continue to be published about the effects of the COVID-19 pandemic on esophagectomy outcomes, the extra data could provide a clearer view of these effects, and therefore provide us with more definitive conclusions. Moreover, the studies included were conducted in high-level institutes of specialized care for esophageal cancer. It could certainly be speculated that the COVID-19 pandemic might have had different results in other institutes, where the creation of green hubs was not possible, and the re-allocation of sources to the more critically ill COVID-19 patients was unproportionally prioritized. Furthermore, the increased hospital stay may be attributed to the fact that several hospitals implemented preoperative COVID-19 testing, with the patient remaining inpatient in quarantine while awaiting for the results. Obviously, this could increase the hospital stay in the pandemic era. This stresses the need to perform further research on whether preoperative COVID-19 or any other virology testing could take place in places other than hospitals (in home settings, for example) to reduce the hospital stay or unnecessary patient hospital visits. Our included studies varied in terms of institution origin, such as the United States of America, the United Kingdom, Italy, and Japan. This reflects the diversity of the included population and may raise the potential selection bias given. Also, there was a significant difference in the included studies’ participants, where the only study with more than 1000 patients was the Maeda et al. Although the heterogeneity was low in some outcomes, the length of hospital stay and age presented high heterogeneity. So, these outcomes should be interpreted with caution while more studies with more patients are needed to achieve more unbiased results. 

## 5. Conclusions

It has been demonstrated that during the COVID-19 pandemic, the major perioperative outcomes of operations for esophageal cancer remain unaffected. Nevertheless, safe surgical operations both for the patients and the surgeons were achieved with the appropriate systemwide policies and patient management.

## Figures and Tables

**Table 1:** Basic characteristics of the included studies.

Author (Year)	Centre	Country	Enrolment Dates	Number of Non-Pandemic Patients (n)	Number of COVID-19 Patients (n)	Male/Female	Age (Mean ± SD)
Khan et al. (2022) [1]	The Johns Hopkins Hospital	USA	3/2019–12/2019 (Control) 3/2020–12/2020 (COVID-19)	190	117	147/43 (Control) 100/17 (COVID-19)	65.1 ± 11.3 (Control) 66.3 ± 10.8 (COVID-19)
Dolan et al. (2021) [10]	Brigham and Women’s Hospital	USA	1/2019–12/2019 (Control) 3/2020–6/2020 (COVID-19)	96	37	78/18 (Control) 32/5 (COVID-19)	-
Borgstein et al. (2021) [11]	Multicenter	Netherlands, Germany, Belgium, and Sweden	10/2019–2/2020 (Control) 3/2020–5/2020 (COVID-19)	168	139	141/27 (Control) 116/23 (COVID-19)	-
Bolger et al. (2021) [12]	Irish esophageal cancer centers	Ireland	4/2019–6/2019 (Control) 4/2020–6/2020 (COVID-19)	53	45	37/16 (Control) 37/8 (COVID-19)	-
Rebecchi et al. (2020) [13]	Multicenter	Italy	3/2019–5/2019 (Control) 3/2020–5/2020 (COVID-19)	60	65	-	66.4 ± 3.5 (Control) 67.7 ± 6.8 (COVID-19)
Milito et al. (2022) [14]	Policlinico San Donato	Italy	3/2019–2/2020 (Control) 3/2020–10/2020 (COVID-19 first cohort) 11/2020–3/2021 (COVID-19 second cohort)	41	29	29/11 (Control) 22/7 (COVID-19)	-
Maeda et al. (2022) [15]	National Clinical Database of Japan	Japan	1/2018–4/2020 (Control) 5/2020–12/2020 (COVID-19)	13,588	3763	-	-
Miyawaki et al. (2022) [16]	National Clinical Database of Japan	Japan	4/2018–3/2020 (Control) 4/2020–3/2021 (COVID-19)	105	52	94/11 (Control) 44/8 (COVID-19)	65.9 ± 8 (Control) 69.2 ± 9.2 (COVID-19)

**Table 2:** Quality assessment of the included studies.

Author, Year	AIM	Inclusion of Consecutive Patients	Prospective Collection of Data	Endpoints Appropriate to the Aim of the Study	Unbiased Assessment of the Study Endpoint	Follow-Up Period Appropriate to the Aim of the Study	Loss to Follow-Up Less than 5%	Prospective Calculation of the Study Size	Total	Risk of Bias
Khan, 2022 [1]	1	2	0	2	2	2	2	0	11/16	Low
Maeda, 2022 [15]	2	2	2	2	2	2	2	0	14/16	Low
Milito, 2022 [14]	2	2	0	2	2	2	2	0	12/16	Low
Miyawaki. 2022 [16]	1	2	0	2	2	2	2	0	11/16	Low
Bolger, 2021 [12]	2	2	0	2	2	0	2	0	10/16	Low
Borgstein, 2021 [11]	1	2	0	2	2	2	2	0	11/16	Low
Dolan, 2021 [10]	2	2	1	2	2	0	2	0	11/16	Low
Rebecchi, 2020 [13]	1	1	0	2	1	2	2	0	9/16	Some concerns

## Data Availability

No new data are created.
